# Photothermal/pH Dual-Responsive Drug Delivery System of Amino-Terminated HBP-Modified rGO and the Chemo-Photothermal Therapy on Tumor Cells

**DOI:** 10.1186/s11671-018-2787-8

**Published:** 2018-11-23

**Authors:** Wei Zhang, Jiamu Dai, Guangyu Zhang, Yu Zhang, Suying Li, Du Nie

**Affiliations:** 1 National and Local Joint Engineering Research Center of Technical Fiber Composites for Safety and Protection, Nantong University, Nantong, 226019 China; 2 School of Textile and Clothing, Nantong University, Nantong, 226019 China

**Keywords:** Graphene oxide, Hyperbranched polymer, Drug delivery, Dual responsive, Chemo-photothermal therapy

## Abstract

In this paper, a simple method to prepare hydrophilic reduced graphene oxide (rGO) was proposed via reducing GO by amino-terminated hyperbranched polymer (NHBP), the as-prepared NrGO could present excellent dispersibility, near infrared (NIR) light absorbance, photothermal conversion ability and stability. Then, the doxorubicin hydrochloride (DOX) was conjugated with NrGO to prepare the drug-loading system, and a pH/photothermal dual-responsive drug delivery behavior was characterized. At acidic environment or under NIR laser irradiation, the drug release rate could be improved, which is beneficial to control release anti-tumor drug in tumor tissues. What is more, the in vitro cell experiments revealed that NrGO was well biocompatible, and in the tumor inhibition part, comparing to the control group without any treatment, DOX@NrGO gained efficient chemo-photothermal synergetic therapy, the inhibition rate of which was much higher than single chemotherapy of released DOX. Therefore, the as-prepared DOX@NrGO obtained great potential application in tumor therapy and an excellent candidate in other biomed applications.

## Introduction

Photothermal therapy (PTT) under near infrared (NIR) irradiation has attracted raising attention for tumor inhibition, due to the little side effect and minimal invasive properties [1]. NIR light (700~1100 nm) penetrate deeper into body tissue without much absorption either any damage to healthy tissue or cells [2, 3]. Thus, under NIR laser irradiation, photothermal agent can raise the temperature in implanted location via its photothermal conversion ability. In addition, the applied photothermal agent requires good biocompatibility, photothermal conversion efficacy, and stability.

For recent years’ researches, variety of materials were designed and prepared to cure tumor tissues as PTT agents, such as precious metal (gold nanorods [4], gold nanoplates [5]), semiconductor nanomaterials (CuS [6], MoS_2_ [7], FeS [8]), organic materials (polydopamine [9], polypyrrole nanoparticles [10]), carbon nanomaterials (carbon nanotube [11], carbon nanoparticles [12], and graphene [13]). As a kind of promising carbon nanomaterial, graphene was widely used in tumor inhibition through PTT method due to its special two-dimensional nanosheets, which obtain ultra-high specific surface area and great potential for high drug loading efficiency [14, 15]. However, reduced graphene oxide (rGO) prepared via normal methods including urea and hydrazine hydrate, hydrothermal process always shows highly hydrophobicity, which is not beneficial to the application in water phenomenon of body tissue [16].

In this case, we proposed a novel idea to use water-soluble polymer with reductive ability to prepare hydrophilic rGO. In our previous work, we synthesized amino-terminated hyperbranched polymer (NHBP) and tried to use it to treat metallic oxide nanoparticles and prepare metal nanospheres, which is highly hydrophilic without obvious agglomeration, such as HBP-modified silver nanoparticles and its application in anti-bacteria field [17, 18].

**Fig. 1:**
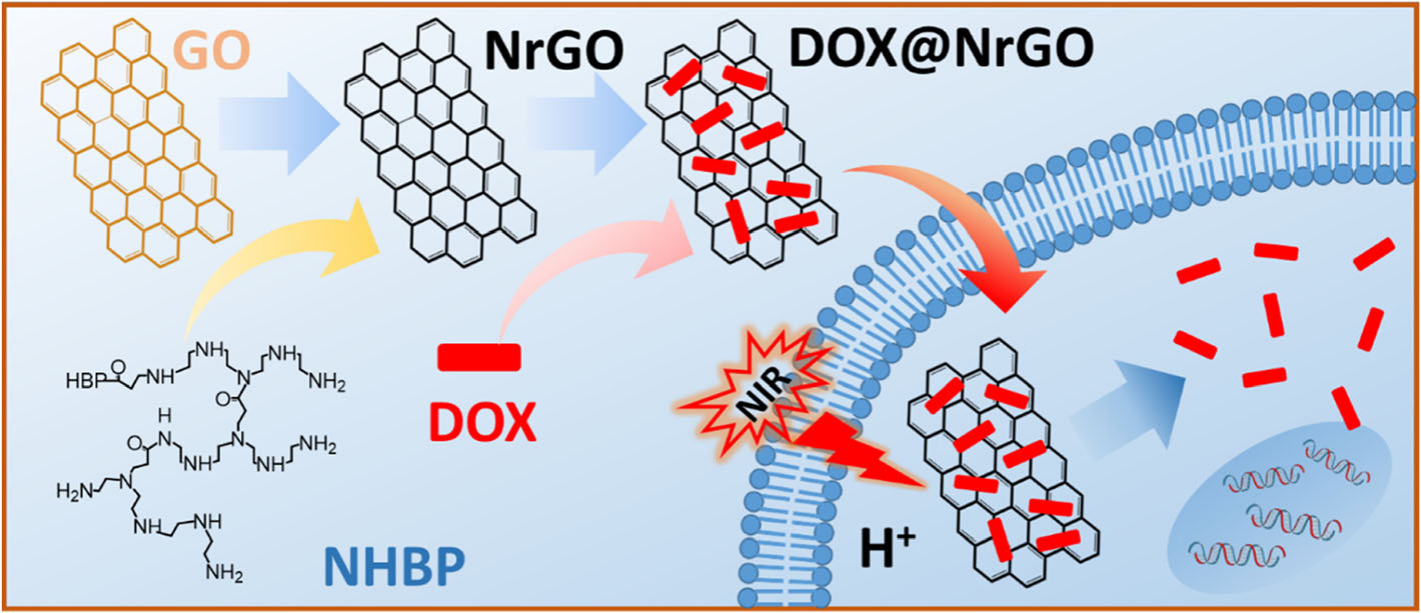
Schematic illustration of preparation and chemo-photothermal therapy of DOX@NrGO

In order to improve the tumor inhibition ability, anti-tumor drugs are usually loaded on photothermal agents to fabricate drug-loaded system [19]. On the one hand, photothermal agent can present PTT effect under NIR laser irradiation. On the other hand, the raised temperature can accelerate drug delivery rate due to the improved molecular movement rate. Thus, drug-loaded photothermal agent can exert chemo-photothermal synergetic therapy effect to tumor inhibition [20, 21]. Herein, we used amino-terminated HBP to prepare hydrophilic rGO (NrGO, Fig. 1), and the physicochemical properties as well as the photothermal ability were characterized. Afterwards, anti-tumor drug (doxorubicin, DOX) was incorporated on NrGO, then the drug delivery behavior under different condition and tumor inhibition efficacy were tested in vitro.

## Methods/Experimental

### Materials

Graphene oxide (GO, 0.8~1.2 nm in thickness and 0.5~5 μm in width) were supplied by XFNANO Co., Ltd. DOX were purchased from HuaFeng United Technology Co., Ltd. Dulbecco’s modified Eagle’s medium (DMEM), fetal bovine serum (FBS), trypsin, penicillin (100 U/ml) and streptomycin (100 μg/ml) were all purchased from Thermo Fisher Scientific Inc. Methyl thiazolyl tetrazolium (MTT), 4′,6-diamidino-2-phenylindole (DAPI) and propidium iodide (PI) were obtained from Beyotime Biotechnology Co., Ltd. All other reagents were purchased from Sinopharm Chemical Reagent Co., Ltd. without further purification.

### Preparation of Amino-Terminated Hyperbranched Polymer (NHBP)

The amino-terminated hyperbranched polymer was synthesized as our previous work [16]. Tetraethylenepentamine (94 ml, 0.5 mol) was added to a 250-ml three-necked round-bottomed glass flask equipped with nitrogen gas protection and magnetic stirring. The reaction mixture was stirred with a heating magnetic agitator and cooled with an ice bath, while a solution of methyl acrylate (43 ml, 0.5 mol) in methanol (100 ml) was added dropwise into the flask. Then, the mixture was removed from the ice bath and left stirring for a further 4 h at room temperature. The mixture was transferred to an eggplant-shaped flask for automatic rotary vacuum evaporation, and the temperature was raised to 150 °C using an oil bath, and left for 4 h until yellowish viscid HBP scale was obtained with a weight average molecular weight about 7759.

### Preparation of NHBP-Reduced GO (NrGO)

GO was first dispersed in deionized water and ultrasonicated mixed with appropriate HBP (weight ratio of GO and NHBP is 1:10, 1:20, and 1:30) for 10 min, kept stirring and reacted under 90 °C for 1 h. Then, the resultant (marked as NrGO-10, NrGO-20, and NrGO-30) was centrifuged and washed with deionized water for three times.

### Preparation of DOX-Loaded NrGO (DOX@NrGO)

The as-prepared NrGO suspension was dispersed in DOX solution with weight ratio of 1:1, and kept stirring for 24 h under room temperature. Then, the composite solution was centrifuged and washed to collect DOX@NrGO.

### Measurements

The surface morphology was characterized via transmission electron microscopy (TEM, JEM-2100, JEOL, Japan). Fourier-transform infrared (FTIR, Nicolet iS10, Thermo Scientific, America) spectroscopy was performed to illustrate the chemical component change between GO and NrGO. All spectra were measured in a wavelength range of 400~4000 cm^−1^ with a resolution of 4 cm^−1^. The surface potential and particle size were investigated through Zeta potential-particle size analyzer (NanoBrook 90plus Zeta, Brookhaven, USA). The absorption of NrGO in NIR region was studied by UV-vis (Evolution 300, Thermo Fisher, USA) with wavelength range of 400~900 nm and resolution of 1 cm^−1^.

The photothermal properties were measured by using a NIR laser device (SFOLT Co., Ltd., Shanghai, China) and a thermocouple thermometer (DT-8891E, Shenzhen Everbest Machinery Industry Co., Ltd., China). The photothermal property of NrGO was measured under 808 nm laser irradiation. The spot area of the laser is about 0.25 cm^2^, and the temperature change of tested sample suspension was monitored in real time. Herein, pure water and GO suspension were applied as control groups: (1) 0.2 ml pure water, GO, and NrGO (NrGO-10, NrGO-20, and NrGO-30) suspension were put in 0.25 ml Eppendorf tube, then NIR laser was irradiated with power density of 1 W/cm^2^ for 5 min; (2) 0.2 ml NrGO-30 suspension with different concentration (100, 200, and 300 μg/ml) was irradiated (1 W/cm^2^) for 5 min; (3) 0.2 ml NrGO-30 suspension (200 μg/ml) was irradiated with different power density (1, 1.5, and 2 W/cm^2^) for 5 min; (4) 0.2 ml NrGO-30 (200 μg/ml) suspension was irradiated (1 W/cm^2^) for three on/off cycles.

The collected DOX@NrGO was divided into three groups for different treatment to investigate the drug delivery behavior: (1) dispersing in PBS solution with pH = 7.4, marked as control group; (2) dispersing in PBS solution with pH = 4.0, marked as acid group; (3) dispersing in PBS solution with pH = 7.4 and irradiated with NIR laser, marked as NIR group. The above three groups (each group was set three parallels) were put in dialysis bag (5 ml) with cut-off molecular weight of 8000, and then put into centrifuge tube with 20 ml corresponding PBS solution. After that, all tubes were put in 37 °C shaker with 100 rpm, 10 ml of PBS solution of each tube was withdrawn at predetermined time points for drug release analysis, and equal volume of corresponding fresh PBS was added back. In addition, the NIR group was treated as that NIR light was irradiated for 5 min after each predetermined time point. All withdrawn solutions were analyzed by UV-vis spectrophotometry, and the drug delivery profile was obtained.

The cytotoxicity of NrGO against tumor cells (HeLa) was investigated by MTT assay. Briefly, HeLa cells were seed in 96-well plates at a density of 5 × 10^3^ cells per well and kept incubating till 80% of the well was covered. Then, the old medium was changed fresh medium with NrGO (3.125, 6.25, 12.5, 25, and 50 μg/ml), the medium without NrGO was set as control group. After incubating for 24 and 48 h, the MTT assay was used to measure the relative cell viability via the Eq. (1):

1 $$ \mathrm{Cell}\kern0.17em \mathrm{viability}\left(\%\right)=\frac{{\mathrm{OD}}_{\mathrm{sample}}}{{\mathrm{OD}}_{\mathrm{control}}}\times 100\% $$

where OD_sample_ and OD_control_ represented the measured absorbance of cells treated with NrGO in different concentration and control group, respectively.

Then, chemo-photothermal synergetic therapy was investigated via treating HeLa cells with DOX@NrGO (3.125, 6.25, 12.5, 25, and 50 μg/ml) under NIR irradiation. After incubating with DOX@NrGO for 4 h, the HeLa cells were irradiated with NIR laser for 5 min and kept incubating for another 20 h. Afterwards, cell viability was tested via MTT assay again. For cells observation, HeLa cells were then stained with DAPI and PI, respectively, and observed under CLSM and fluorescence microscope.

## Results and Discussion

### Physical and Chemical Characterization

**Fig. 2:**
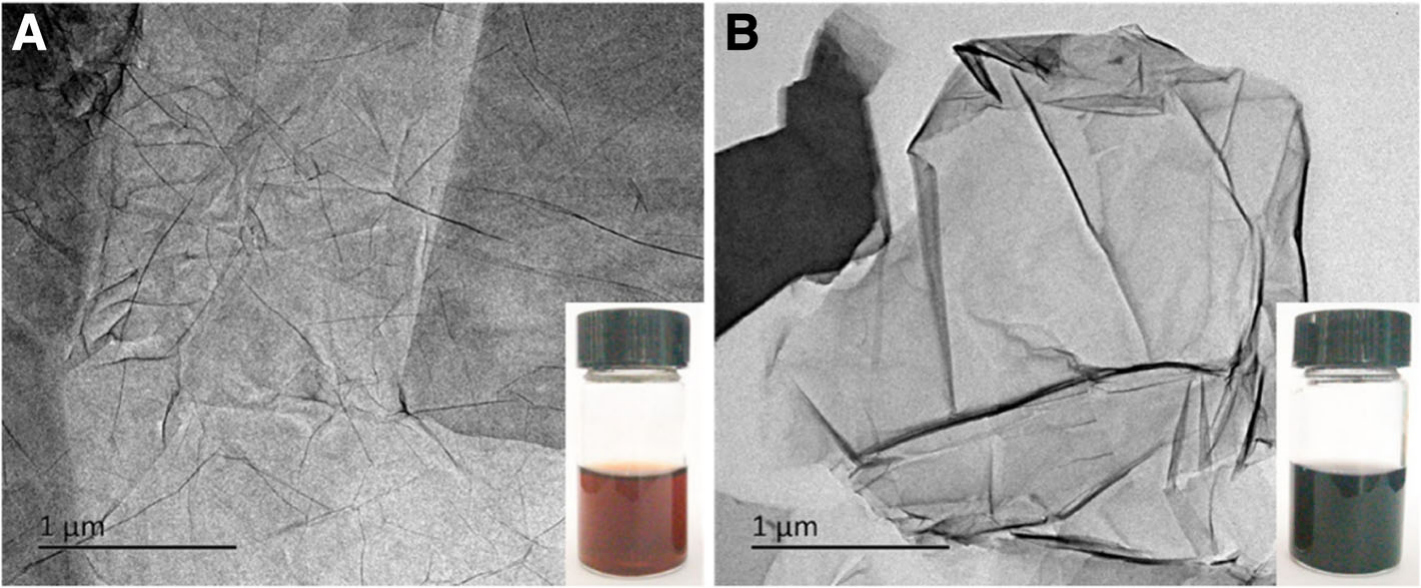
TEM images of GO (**a**) and NrGO (**b**). The inset picture is optical photograph corresponding sample dispersion with concentration of 1 mg/ml

**Fig. 3:**
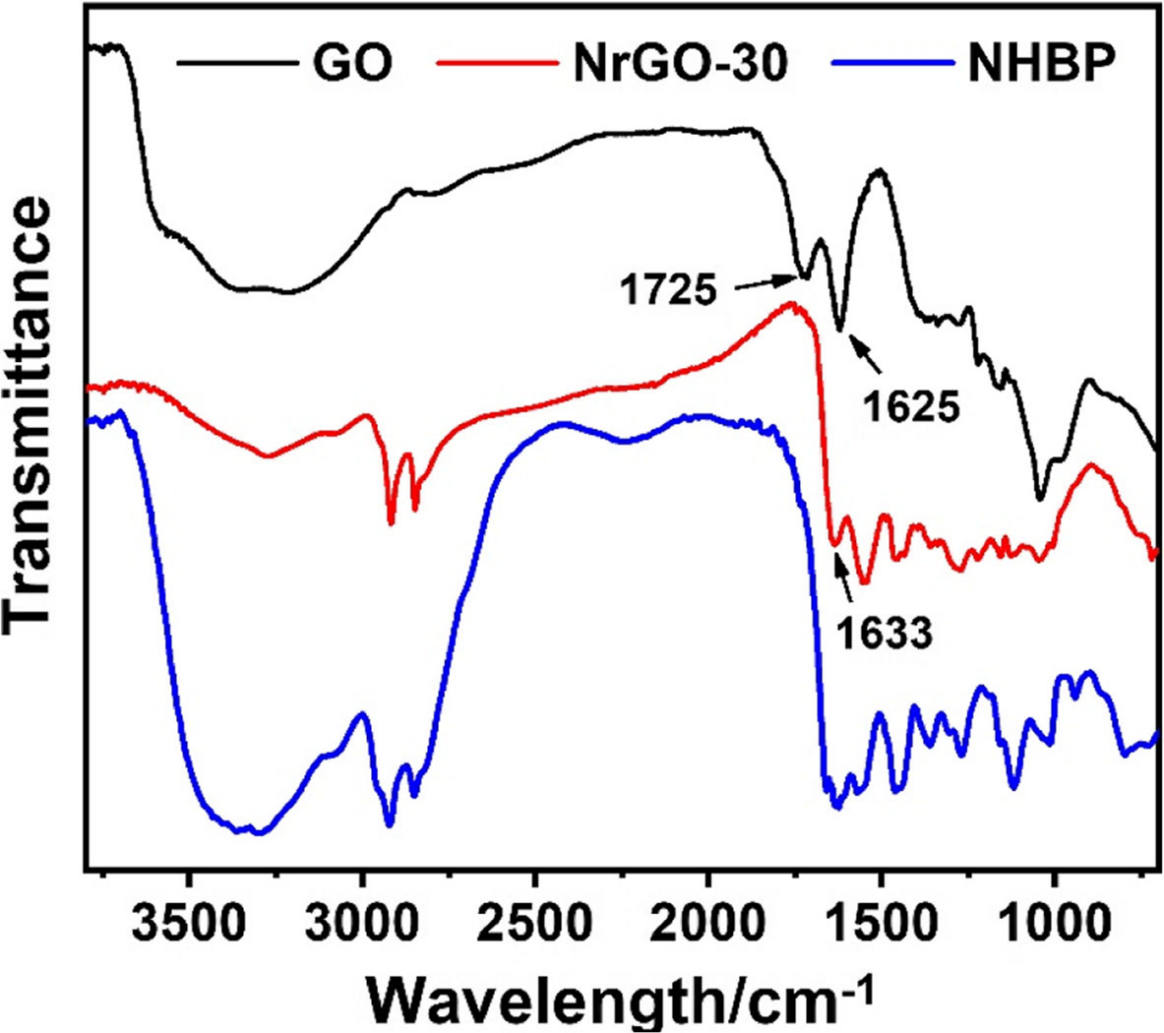
FT-IR spectra of GO, NrGO, and NHBP

**Fig. 4:**
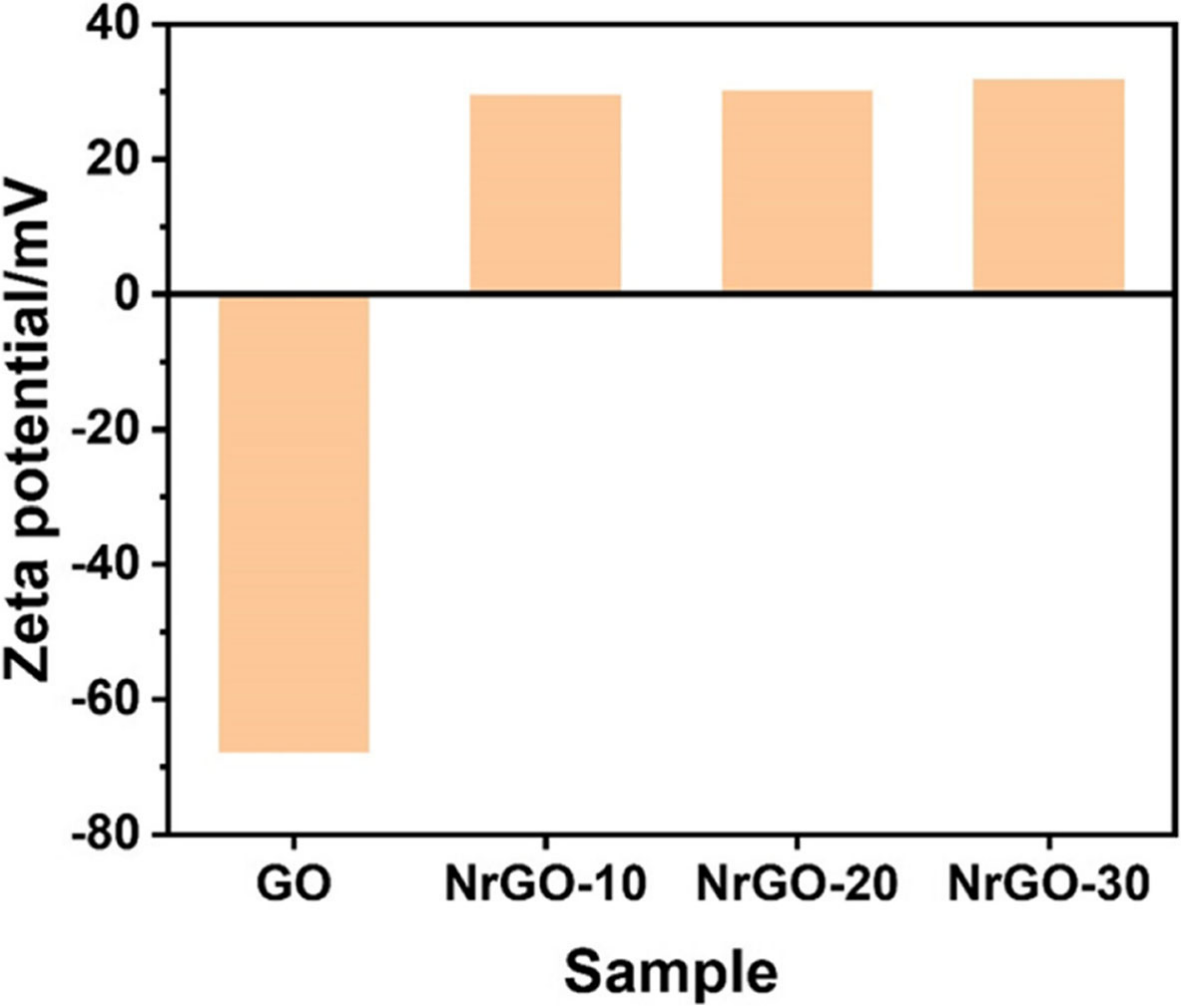
Zeta potential test of GO and NrGO samples

**Fig. 5:**
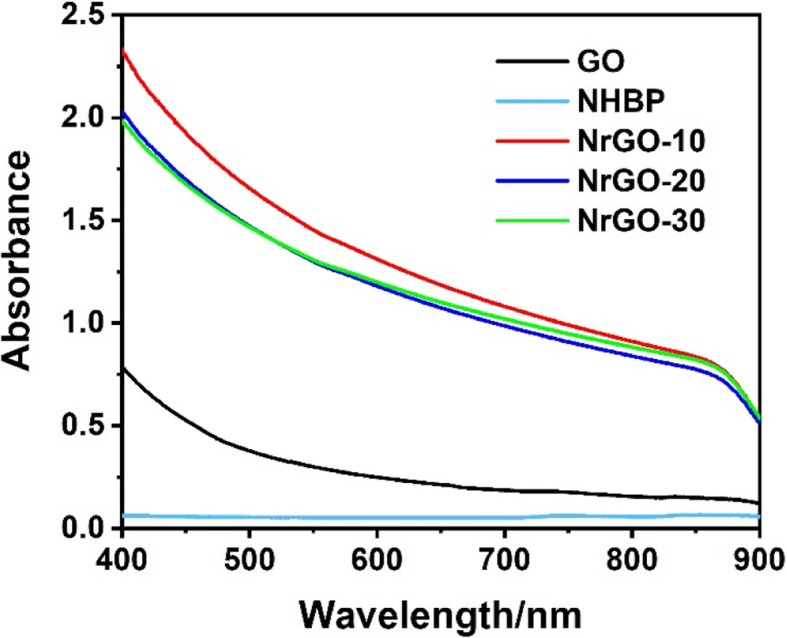
UV-vis-NIR spectra of GO, HBP, and NrGO samples

**Fig. 6:**
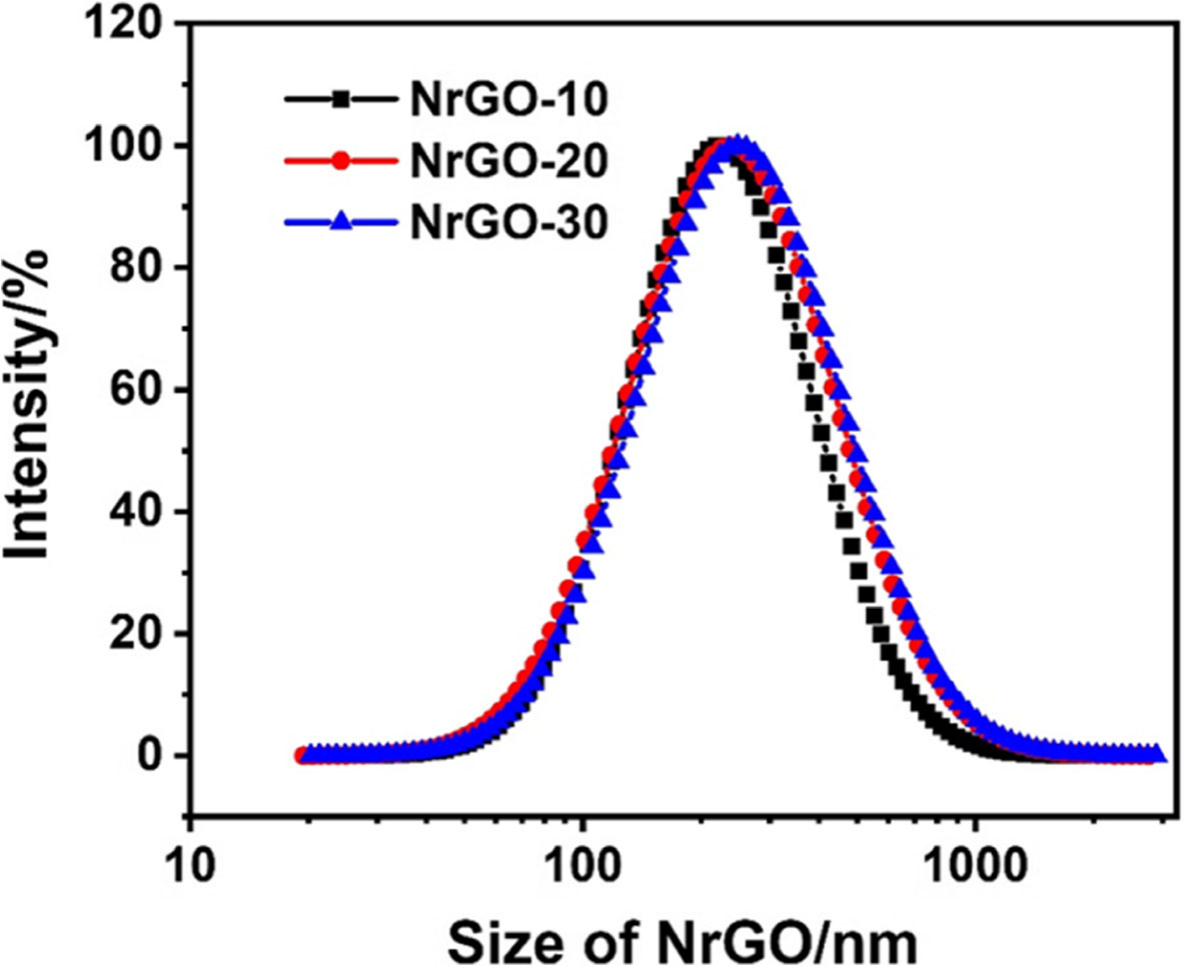
Nanosize measurement of NrGO samples

After reacting with NHBP, the GO solution turned to black from brown, indicating that GO was successfully reduced to rGO and dispersible in water. As shown in Fig. 2a, b, TEM images of GO and NrGO-30 were exhibited respectively, while no obvious crispation or agglomeration was discovered on NrGO, revealing the HBP treatment would not cause any morphology change at reducing reaction. Based on the FT-IR spectra in Fig. 3, the transmittance curve of NrGO-30 was very similar to that of NHBP. Significantly, the peak at 1725 cm^−1^ of GO was disappeared after reducing reaction, which was suggested to be vibration absorption of C=O from carboxy group [22]. According to the molecular structure of amino-terminated NHBP, the reductive amino group reacted with GO and new FT-IR peak was generated at 1633 cm^−1^, which supposed to be C-N from amido bond. The zeta potential result was presented in Fig. 4, obviously, all NrGO samples were positive potential while GO was negative, indicating that the carboxy group of GO was reacted with amino group from HBP. UV-vis-NIR spectra (Fig. 5) was used to illustrate the NIR absorption of NrGO; the curves of NrGO samples with different raw materials ratio showed similar trend with high absorption at NIR region, which is beneficial to the application in PTT. Whereas, GO and HBP solution barely showed absorption at NIR region, suggesting the successful fabrication of photothermal agent from GO and NHBP. In addition, the nanosize of NrGO was also measured (Fig. 6), which did not show obvious change with NHBP ratio raising.

### Photothermal Properties Measurement

**Fig. 7:**
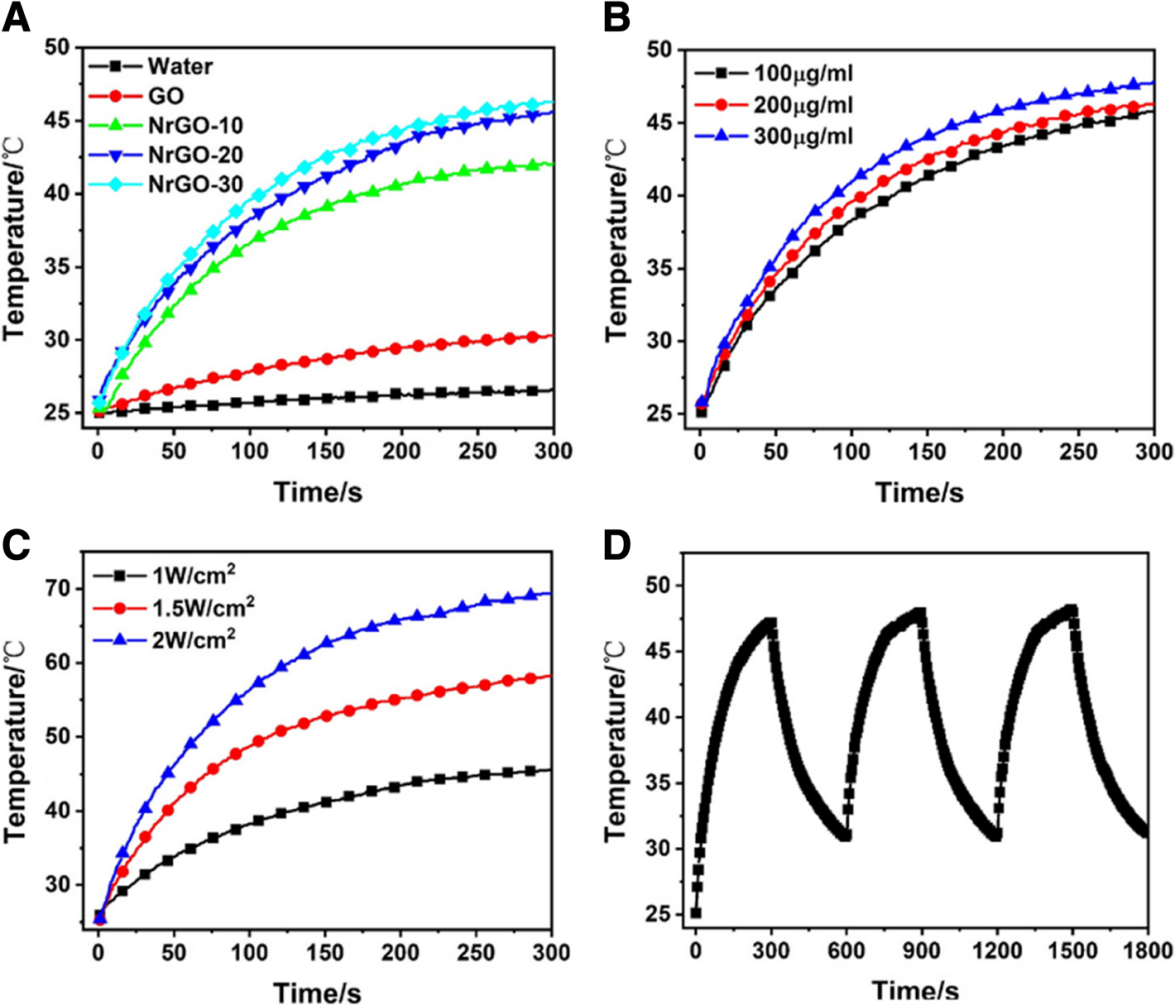
Photothermal properties measurement. **a** Heating curves of water, GO, and NrGO samples (200 μg/ml) under 808 nm laser irradiation (1 W/cm^2^). **b** Heating curves of NrGO-30 with different concentrations under 808 nm laser irradiation (1 W/cm^2^). **c** Heating curves of NrGO-30 (200 μg/ml) under 808 nm laser irradiation at different power density. **d** Temperature change curve of NrGO-30 (200 μg/ml) under 808 nm laser irradiation for cycle irradiation of three times (1 W/cm^2^)

**Fig. 8:**
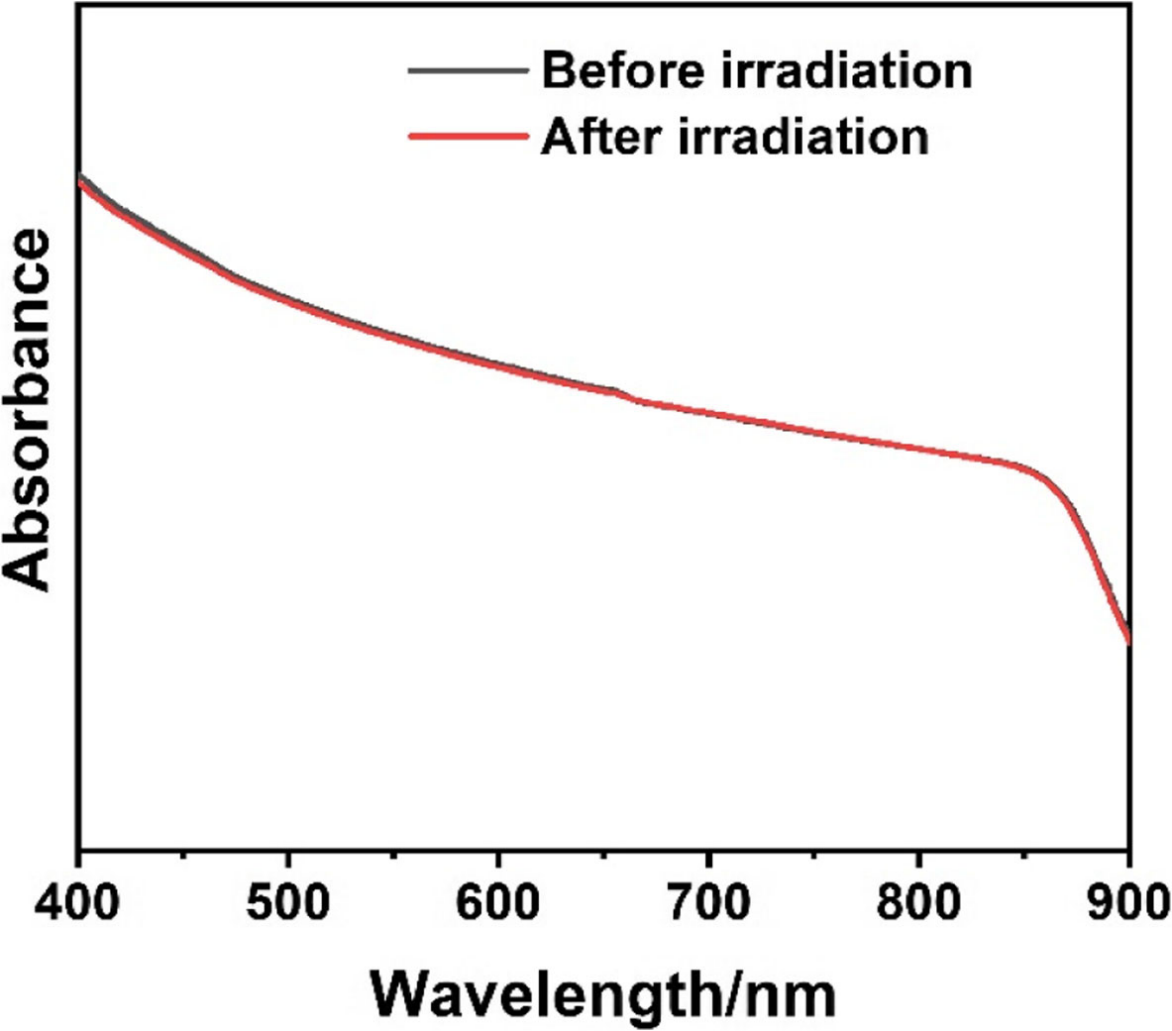
UV-vis-NIR spectra of NrGO before and after NIR laser irradiation

Based on the obtained NrGO, the photothermal properties were investigated under 808 nm laser irradiation. As shown in Fig. 7, the heating curves of water, GO, and NrGO presented different trend. The temperature of pure water almost showed no growth, and GO only raised lower than 5 °C, while NrGO improved up to 40 °C and NrGO-20 and NrGO-30 even reached higher than 45 °C. NrGO could absorb NIR laser to trigger photothermal behavior, and the photothermal conversion efficacy was improved with HBP ratio raising; therefore, NrGO-30 was chosen to complete the following investigation. As illustrated in Fig. 7b, c, the reached temperature was raised with NrGO concentration or laser power increase, and the latter factor affected more strongly. 41–43 °C was proved to be appropriate in tumor cells inhibition with little negative effect on normal cells; thus, the prepared NrGO could meet the requirement of PTT in a low dosage and laser powder. Then, the photothermal stability was tested and showed in Fig. 7d, there is no obvious difference after three on/off cycles. Thus, NrGO obtained great photothermal properties in NIR region. To confirm the absorption stability of NrGO before and after NIR laser irradiation, the UV-vis spectra were showed in Fig. 8. Obviously, the curve did not change after NIR irradiation, revealing the NIR irradiation would not affect the absorption of NrGO.

### Drug Delivery Behavior Test

**Fig. 9:**
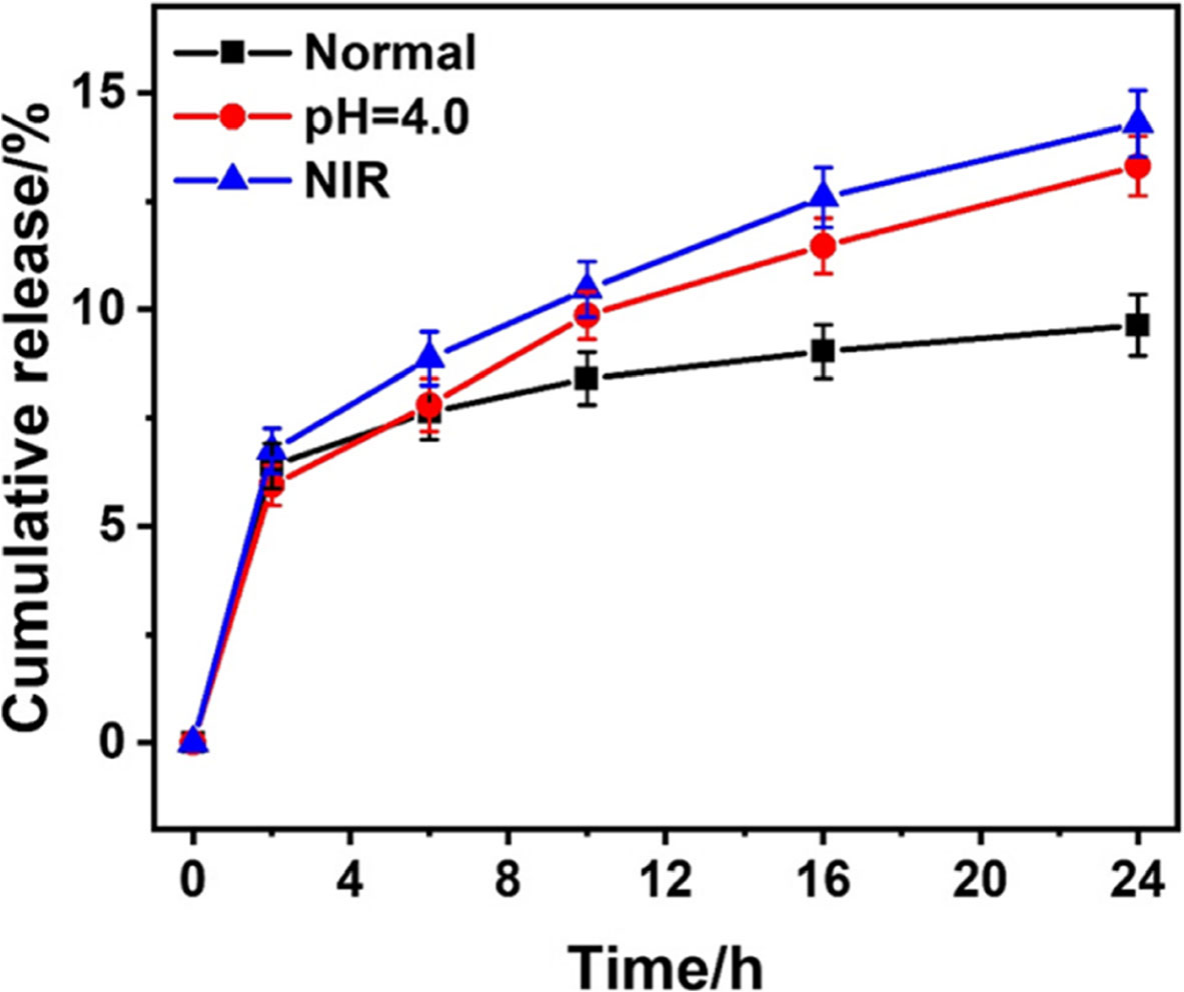
In vitro drug release profiles of DOX@NrGO under different condition

After DOX was loaded on NrGO, the drug delivery experiment was carried out. Due to the faintly acid environment of tumor tissue, the influence of NIR irradiation and pH were both studied. Herein, PBS with pH of 7.4 or 4.0 was applied to mimic the normal or tumor tissue, respectively. As shown in Fig. 9, the drug delivery rate was obviously accelerated under low pH and NIR irradiation. On the one hand, the amino group of NrGO would be ionized under low pH value, then repulsive force between DOX and ionized amino groups would be improved under low pH condition, which accelerated drug delivery and showed pH sensitivity. Besides, the good solubility of DOX in low pH condition could also increase the drug delivery rate [23]. On the other hand, with the NIR laser irradiation, the local temperature was raised and molecular motion rate was speeded up. Thus, the DOX@NrGO was pH/photothermal sensitive in drug delivery behavior, which is beneficial to control the drug delivery rate in tumor tissue and exerted chemo-photothermal synergetic therapy.

### Cytotoxicity of NrGO

**Fig. 10:**
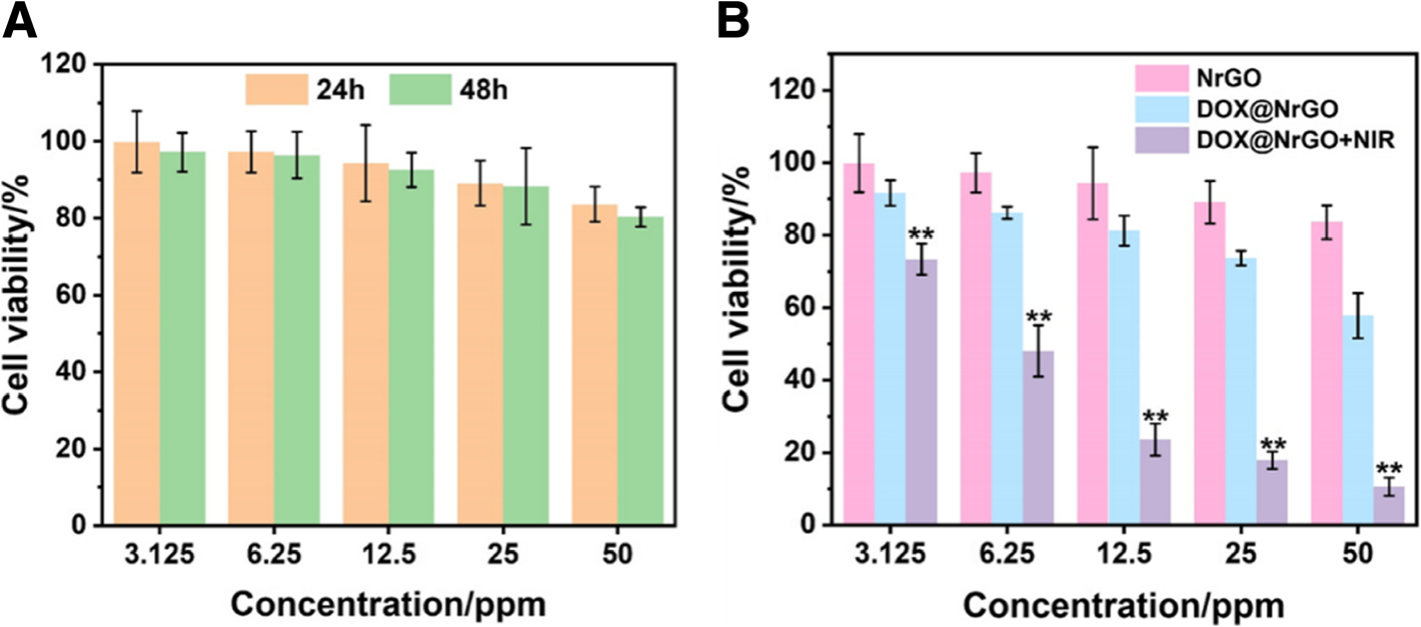
**a** Cytotoxicity test of NrGO at different concentration for 24 h and 48 h. **b** Tumor cell inhibition investigation of DOX@NrGO with different treatment

Biocompatibility is the basic required property in biomaterials; thus, the cytotoxicity of NrGO with different concentration was initially tested during in vitro experiment via MTT assay. As shown in Fig. 10a, the MTT assay results of 24 h indicated that the cell viability remained more than 80% when NrGO concentration reached 50 μg/ml, which can prove that NrGO was well biocompatible and regarded as promising biocompatible PTT agent in tumor inhibition.

### Synergetic Inhibition of DOX@NrGO on Tumor Cells

Based on the biocompatibility of NrGO, the tumor inhibition efficacy of DOX@NrGO was studied in vitro. In order to exam the influence of photothermal behavior, NIR laser was irradiated on corresponding tumor cells for 5 min with power density of 0.5 W/cm^2^. As demonstrated in Fig. 10b, when tumor cells were treated with DOX@NrGO for 24 h, the viability decreased obviously with concentration increase, revealing the released DOX could inhibit tumor cell proliferation. Moreover, the viability decreased much more rapidly when NIR irradiation was applied as well, indicating the raised temperature and DOX release rate could play chemo-photothermal synergetic therapy.

**Fig. 11:**
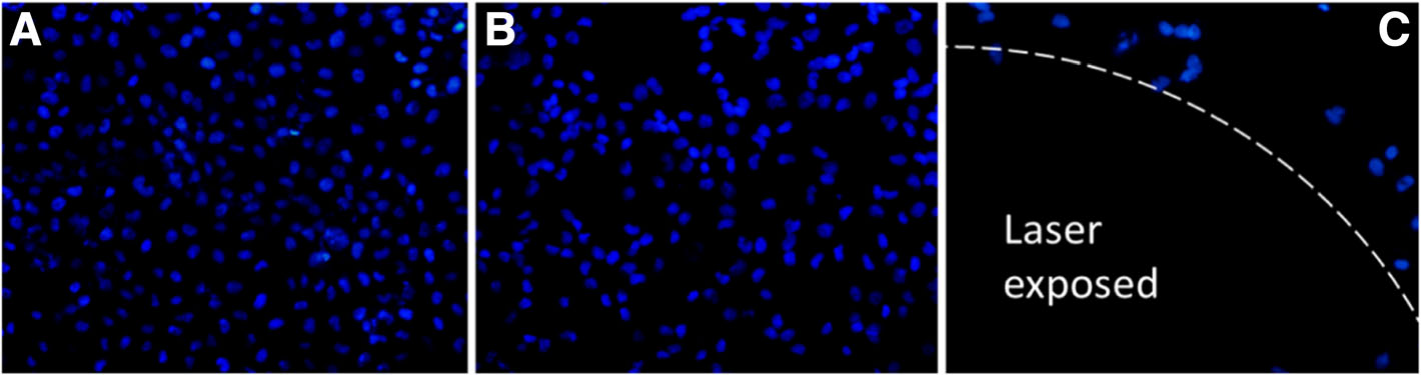
CLSM images of DAPI (blue) stained cell nuclei after treating with NrGO (**a**), DOX@NrGO (**b**), and DOX@NrGO+NIR (**c**). (× 400)

After staining with DAPI, the cells were observed under confocal laser scanning microscopy (CLSM), the nucleus was stained to blue and the images of different treatment were displayed in Fig. 11a–c, respectively. The cells cultured with NrGO were overspread (Fig. 11a) on the cultural plate with a large amount, while the number decreased when treated with DOX@NrGO (Fig. 11b), revealing the released DOX could inhibit the tumor proliferation. Significantly, tumor cells at NIR laser exposure region were efficiently destroyed and fell off, which resulted in a dark area on the image (Fig. 11c).

**Fig. 12:**
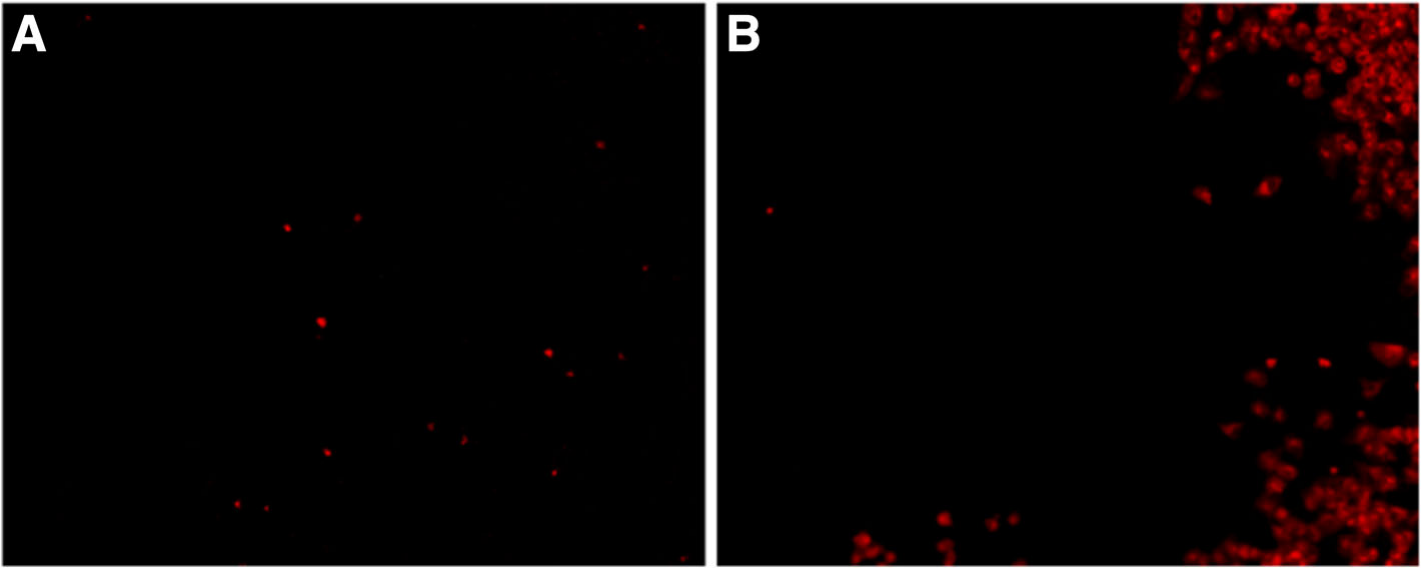
PI staining of tumor cells with different treatments. **a** Control. **b** DOX@NrGO+NIR

In addition, PI was applied to observe tumor cell inhibition after chemo-photothermal synergetic treatment, which is a kind of small molecular dye to stain dead cell to red fluorescence. As shown in Fig. 12, seldom dead cells (red point in the image) were observed in Fig. 12a when no treatment was carried out, while after chemo-photothermal treatment, tumor cells out of exposure region suffered from the damage of DOX and high temperature to further reduce cell viability (Fig. 12b). According to the above results, DOX@NrGO was proved to be a desired candidate for tumor therapy.

## Conclusions

In summary, novel hydrophilic NrGO was designed and successfully prepared via simple reaction of GO and amino-terminated HBP. Varied characterization showed that NrGO obtained stable and outstanding photothermal property. After DOX loading, the drug delivery presented pH and photothermal dual-responsive behavior, which could be accelerated at low pH value and NIR irradiation. In addition, in vitro cytotoxic experiment result showed that the as-prepared NrGO was well biocompatible. Due to the advantage, tumor cells could be effectively inhibited based on chemo-photothermal synergetic therapy, and anti-tumor drug-loaded NrGO obtained promising application in tumor therapy.

## Abbreviations

CLSM: Confocal laser scanning microscopy
DAPI: 4′,6-diamidino-2-phenylindole
DOX: Doxorubicin
DOX@NrGO: DOX-loaded NrGO
FTIR: Fourier-transform infrared
GO: Graphene oxide
HBP: Hyperbranched polymer
MTT: Methyl thiazolyl tetrazolium
NHBP: Amino-terminated HBP
NIR: Near infrared
NrGO: Amino-terminated hyperbranched polymer reduced graphene oxide
PI: Propidium iodide
PTT: Photothermal therapy
rGO: Reduced graphene oxide
SEM: Scanning electron microscopy
TEM: Transmission electron microscope
